# Training Data Recorders with a Manual to Take High-Quality Trachoma Photographs in South Sudan

**DOI:** 10.4269/ajtmh.26-0213

**Published:** 2026-07-14

**Authors:** Madison Bearden-Muse, Nicholas A. Presley, Stephen Ohidor, Tania A. Gonzalez, Lochebe Boniface, Stella Abuba, Shamika V. Chavda, Reida Konga, Fisseha Admassu, E. Kelly Callahan, Yak Yak Bol, Angelia M. Sanders, Scott D. Nash

**Affiliations:** ^1^ The Carter Center, Atlanta, Georgia, USA;; ^2^ The Carter Center, Juba, South Sudan;; ^3^ Emory University, Atlanta, Georgia, USA;; ^4^ Department of Ophthalmology, University of Gondar, Gondar, Ethiopia;; ^5^ South Sudan Ministry of Health, Juba, South Sudan

## Abstract

When a country has reached a low trachoma prevalence, it can be difficult to reliably train survey graders to accurately grade trachoma signs. Conjunctival photography provides an opportunity to capture images for grading. Recently, a training manual targeting survey data recorders was published. In July 2023, this manual was used during a photography training, after which certified trainees served as data recorders and photographers during the Enhancing the ‘A’ in SAFE (ETAS) study in South Sudan. Five of 10 trainees passed the training and became certified. These individuals took 7,064 smartphone photographs (two photographs per eye) of 1,766 children during the ETAS study. Certified photograph graders at the Gondar Grading Center in Ethiopia determined that nearly all eyes (>99%) had at least one gradable photograph. The new manual successfully facilitated the training of data recorders to take gradable conjunctival photos and will be an important tool for trachoma programs.

## INTRODUCTION

Trachoma is caused by repeated infections of the *Chlamydia trachomatis* bacterium.^1^ During surveys to assess trachoma prevalence, trained/certified graders examine individuals for clinical signs, including trachomatous inflammation-follicular (TF) among children ages 1–9 years. Grader training has traditionally relied on the availability of children with and without TF to allow for intragrader reliability and certification assessments. However, in areas with low trachoma endemicity, it can be difficult to find enough children with TF for training and certification. Because of these logistical challenges, trachoma programs have recently begun to rely on certifying graders using photographic images of only the conjunctivae.^2^ A clear next step for trachoma programs is to evaluate the incorporation of conjunctival photographs as part of surveys themselves.

In 2024, the International Coalition for Trachoma Control (ICTC) published a photography training manual targeting survey recorders without prior experience to use a smartphone camera to capture conjunctival images.^3^ Although previous trachoma research has relied on digital cameras, it is likely that smartphones are easier to manage in the field and cheaper than traditional camera models.^4^ In 2023, the Republic of South Sudan’s (RSS) Trachoma Control Program (TCP) used a prepublished version, with the goal of assessing the feasibility of the manual in a real-world setting and to assess the gradability of photographs taken by those trained.

## MATERIALS AND METHODS

Conjunctival photography was included during the postintervention follow-up survey of the “Enhancing the ‘A’ in SAFE (ETAS)” trial (NCT05634759) in Kapoeta North County in 2023.^5^ The survey design approximated a traditional trachoma survey in terms of sampling, sample size, and field procedures.^6^ A 4-day training course was conducted by TCP staff with experience in conjunctival photography. Training materials, excluding the trachomatous trichiasis sections, were created and adapted for the study based on the draft ICTC *Training Photographers for Trachoma Prevalence Surveys: A Manual on Preferred Practices*.^3^

The training consisted of a preliminary evaluation followed by three days of in-class learning, one day of field practice, and certification activities. Ten recorder trainees, three women and seven men ranging in age from 25 to 35 years, were recruited and assessed, based on a scale of 0 to 10, on previous experience, which included the number of instances where they had similar work experience, survey recorder experience, and conjunctival photography experience. Similar work experience equated to working in community-based activities, such as drug administration, surveys, and other programmatic activities. Recorder experience corresponded to instances where electronic data capture was used, which were almost exclusively trachoma surveys.

The preliminary evaluation, adapted by TCP, observed applicants’ competencies through four tests (each based on a scale of 1 to 5) (Supplemental File 1). The near vision test assessed the trainee’s ability to see sharp contrast letters at a near distance (approximately 40 cm) and be able to read up to the 20th line. During a series of simulated practice interaction scenarios, trainees were assessed on the comfortability of smartphone use, working with mother and child, and working in group settings. Scores on the four tests were aggregated, and trainees scoring 15 or higher proceeded with the training.

The classroom sessions consisted of 1) overview of trachoma and ocular anatomy, 2) introduction to camera and care of equipment, 3) instructional on quality photography, 4) survey protocol, and 5) four practice photography sessions. During the first session, trainees photographed a conjunctivae-shaped diagram on a piece of fruit per the manual, and the last three sessions involved taking photographs of each other’s eyes. Trainees then progressed to the field practice where they photographed the conjunctivae of up to five children, and supervisors evaluated the trainees’ performance on a three-step Likert scale (poor, average, done well). This evaluation was followed by an examination, where each trainee photographed both eyes of six additional children while being observed by training staff who assessed their field procedures and the photograph gradability. Supervisors scores were calculated on the percentage of procedures marked “satisfactory,” the percentage of gradable photographs (*n* = 24), and the percentage of eyes (*n* = 12) with at least one gradable photograph. To have a passing score, the ICTC manual indicates that trainees should have successfully taken images of six children, had fewer than 10 unsatisfactory marks, and had no ungradable images.^3^ To be certified, trainees needed to complete the course and have taken gradable photographs of six children in the presence of the trainer during the final examinations.

### Data collection.

After the training, certified trainees served as survey recorders in the ETAS study, which included conjunctival photography within Open Data Kit.^5^ Using Google Pixel^®^ 6 Pro phones without attachment, two photographs per eye were taken of all study children ages 6 months to 9 years. After data collection, three different trained photograph graders from the Gondar Grading Center (GGC) in Amhara, Ethiopia, in partnership with the RSS-TCP, independently graded the photographs for image quality (“good,” “poor but acceptable,” and “ungradable”) on a custom-built database using the R Shiny v. 1.11.1 (https://shiny.posit.co/) application.^7^ Summary statistics were calculated to describe the proportion of images in each quality category using R (R Development Core Team, 2025). Information on why a photograph was designated as “poor but acceptable” or “ungradable” was not captured in the grading system; however, a sample of photographs with these designations were reviewed to understand the issues with quality. Trachoma outcomes generated from photographic grading will be reported elsewhere.^5^

## RESULTS

### Photography training.

**Figure 1.**
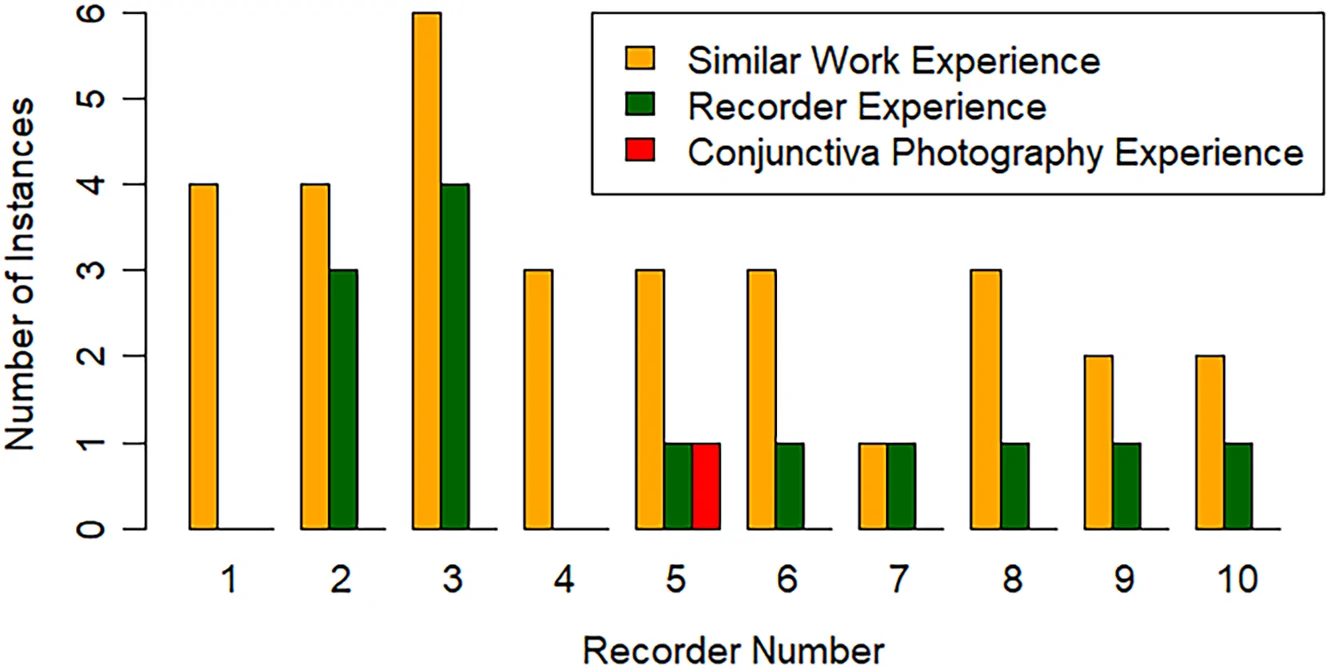
Summary of previous survey experience per recorder prior to training, Enhancing the ‘A’ in SAFE Study, Kapoeta North, Republic of South Sudan, 2023. All recorders reported similar work experience with a range of one to six previous instances, eight recorders reported previous recorder experience with a range of one to four instances, and one recorder reported one instance of conjunctival photography experience.

Ten applicants were recruited to participate in the photography training with the goal of including five recorders/photographers as part of the trachoma survey. Each trainee had participated in similar work and/or had recorder experience prior to the training; however, only one trainee had prior conjunctiva photography experience (Figure 1). All 10 applicants passed the preliminary evaluation (Table 1). Once the classroom sessions and field practice were completed, the 10 trainees took the first certification exam. None of the 10 photographers passed the first certification round. Therefore, among the trainees with the top scores, five were given a “conditional pass” and were invited to continue practicing and allowed to retake the certification, whereas the five with the lowest scores were dismissed. After additional practice, four out of five passed a second certification attempt, and the fifth passed after additional practice and a third certification round (Table 1).

**Table 1:** Summary of evaluations and results from conjunctival photography training and general recorder training, enhancing the ‘A’ in SAFE Study, Kapoeta North, Republic of South Sudan, 2023

Recorder Number	Number	1	2	3	4	5	6	7	8	9	10
Preliminary evaluation	Preliminary assessment total (*n* = 4)*	19	18	15	18	20	18	17	18	16	19
	% Possible points achieved	95	90	75	90	100	90	85	90	80	95
Field practice	Number of children examined	3	5	5	3	3	3	3	3	4	4
	% Responses marked as done well or average	100	100	96	100	100	100	100	56	100	90
Certification round 1	Number of children examined	6	6	6	6	6	6	6	6	6	6
	Average age of children in years	3.3	3.0	3.1	4.2	2.3	3.8	3.7	5.2	4.2	3.0
	% Responses marked as “Satisfactory”	84	96	73	86	92	89	94	70	80	93
	% Gradable photos (*n* = 24)^†^	52	35	42	44	46	33	23	31	29	29
	% Eyes with at least 1 gradable photo (*n* = 12)^‡^	75	58	67	58	58	54	38	38	50	50
	Conditional certification?	Yes	Yes	Yes	Yes	Yes	No	No	No	No	No
Certification round 2	Number of children examined	6	6	6	6	6					
	Average age of children	2.8	5.0	5.7	3.7	3.3					
	% Responses marked as “Satisfactory”	93	69	97	90	97					
	% Eyes with at least 1 gradable photo (*n* = 12)^§^	67	100	100	100	100					
	Pass certification?	No	Yes	Yes	Yes	Yes					
Certification round 3	Number of children examined	6									
	Average age of children	7.7									
	% Responses marked as “Satisfactory”	89									
	% Eyes with at least 1 gradable photo (*n* = 12)^§^	100									
	Pass certification?	Yes									

* Number of preliminary assessments conducted.

^†^ Calculated as: ([number of gradable photos by grader A plus number of gradable photos by grader B]/2)/24.

^‡^ Calculated as: ([number of eyes with at least one gradable photo by grader A plus number of eyes with at least one gradable photo by grader B]/2)/12.

^§^ One to one grader reviewed the photos from Certification Round 2.

### Survey and trachoma grading.

During the ETAS study, newly certified photographers took 7,064 photographs (two photographs per eye) of 1,766 children ages 6 months to 9 years. Once the survey was completed, photographs were sent to the GGC to be graded for image quality. Grades were generated at the eye level (*n* = 3,532), with graders reviewing both photographs to determine the grade. The GGC scoring demonstrated that nearly all eyes (>99%) were determined to have at least one gradable photograph (Table 2). Between the three photograph graders at GGC, all three agreed that photograph quality was “good” for 41.6% (1,470/3,532) of the eyes, and two of three graders agreed quality was “good” for a further 33.8% (1,193/3,532) of eyes. Two eyes were deemed “ungradable” and 7.1% (252/3,532) were considered “poor but acceptable” quality by all graders. Among the images classified as “poor but acceptable,” the observed photography issues had to do with glare, positioning, and blurriness of the conjunctiva; however, blanching was also observed in the eyelid eversion. The two photographs deemed “ungradable” were not images of the conjunctiva. Randomly selected graded photographs are shown in Supplemental Figure 1.

**Table 2:** Summary of photograph gradability agreement types across three computer graders, enhancing the ‘A’ in SAFE Study, Kapoeta North, Republic of South Sudan, 2023

Photograph Grading Agreement	*n*	Percent
All 3 graders reported “Good”	1,470	41.6
2 out of 3 Graders reported “Good”	1,193	33.8
1 out of 3 Graders reported “Good”	615	17.4
All 3 Graders reported “Poor but acceptable”	252	7.1
Ungradable	2	0.1

## CONCLUSION

This study demonstrated that data recorders could be trained to take gradable photographs with the newly published ICTC manual. Overall, the ICTC manual provided clear but adaptable curricula to facilitate photography training within a programmatic setting. Using the manual, five recorders with little experience in conjunctival photography were trained and certified to work in a survey setting and further took more than 3,000 photographs deemed gradable by a center specializing in trachoma grading.

Conjunctival photography has been used in trachoma research for years. A systematic review demonstrated that photographic grading compares well with field grading, particularly when digital cameras are used.^4^ More recent studies have begun to rely on smartphone photography, and some studies suggest that newer phone models perform as well as or better than digital single-lens reflex cameras.^8^^–^^12^ Photography as part of surveys has been shown to be acceptable by study teams and populations.^13^^,^^14^ Further, the use of photographs in lieu of field grading has already been deployed in areas where grader certification was deemed impossible.^11^ Grading centers such as the GGC in Ethiopia should allow for programs to receive grading results in time to make intervention decisions, and machine learning technologies could possibly provide a TF decision on the phone while still in the field.^7^^,^^15^^,^^16^ Future studies should seek to capture more nuanced data on image quality within software systems to allow for useful feedback for photographers and graders. With high-quality training, quality control, and low levels of ungradable photographs, smartphone photography represents a promising tool for trachoma programs as they reach elimination thresholds.

The TCP encountered challenges while implementing the photography training. One issue was that there were more recruited trainees (*n* = 10) than available phones (*n* = 7) and graders who could evert eyelids (*n* = 5). This situation led to the rotation of graders and phones during exercises. Another challenge was the low number of children included in the field practice prior to the certification exams, which may have been a primary factor in the low scores on the first exam. Including more practice sessions with more children during the training (which should be specified more clearly in the manual for better results) is recommended. Although successful in the end, these issues led to the training running longer than anticipated. In a recent study, Aguwa et al. also used a prepublished version of the ICTC manual with fewer trainees and reported that all trainees passed and went on to take high rates of gradable images.^9^ The South Sudan experience suggests that it would be beneficial to have an adequate number of trainers and trained graders prior to starting the training.

The training’s success in South Sudan, a country with significant challenges and a high prevalence of trachoma, demonstrates the potential for photography to be used effectively in programmatic settings.

## Supplemental Materials

10.4269/ajtmh.26-0213 Supplemental Materials
