# Supplementary material for: Training Data Recorders with a Manual to Take High-Quality Trachoma Photographs in South Sudan
Source: Am J Trop Med Hyg. 2026 Jul 14;115(3):464–7. doi: 10.4269/ajtmh.26-0213 (PMC13539812; doi:10.4269/ajtmh.26-0213)
Supplement: Supplementary file 1 [file tpmd260213.SD1.pdf]

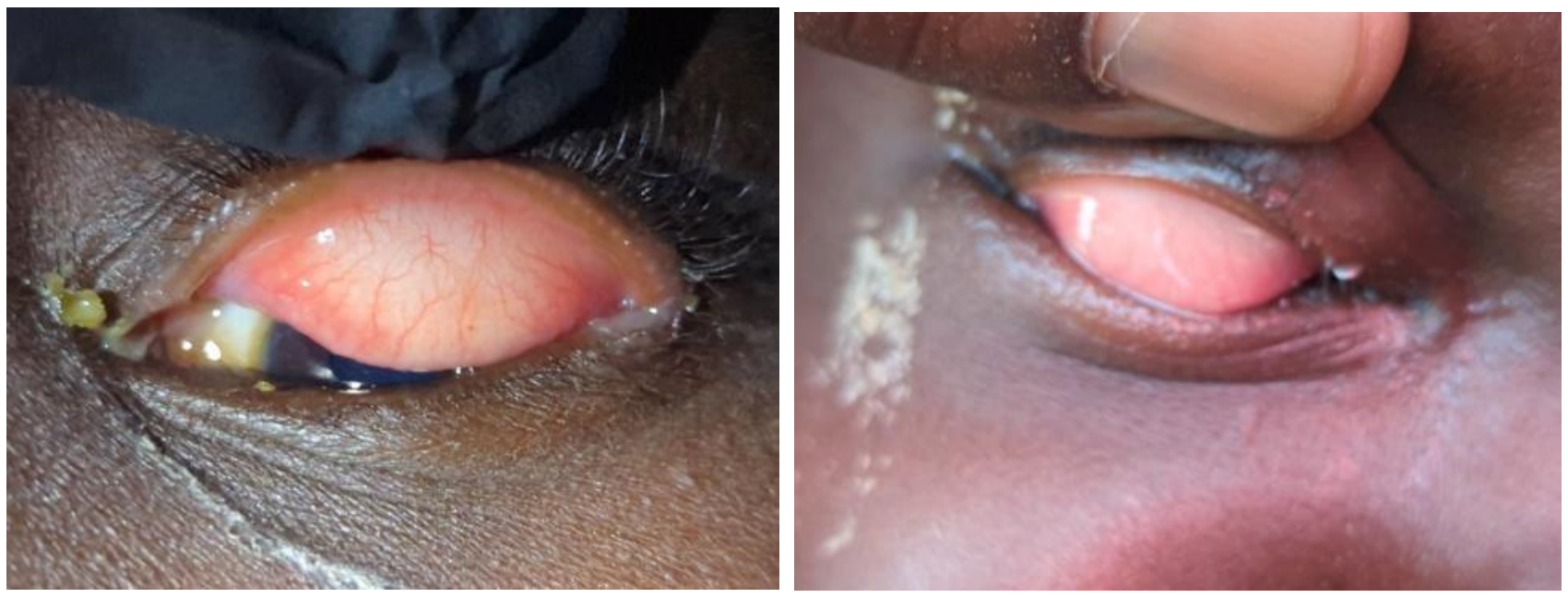

“Good” “Poor but acceptable”

Supplemental Figure 1: Examples of “Good” and “Poor but acceptable” quality images agreed upon by all three computer graders, ETAS Study Kapoeta North, Republic of South Sudan, 2023. The image on the left was considered good by the computer graders and is clearer, more focused and better positioned. The image on the right was considered poor but acceptable and it is slightly out of position and is less clear.

PRELIMINARY ASSESSMENT FOR PHOTOGRAPHER TRAINEES - 2023

Examiner: Please observe the photographer trainees in all the following components and indicate whether each component is performed satisfactorily or unsatisfactorily.

**Applicant Name**	**Near Vision** ***(Pass / no pass)***	**Smartphone Use** ***(score 1-5)***	**Working with Mothers and Children** ***(score 1-5)***	**Working in groups** ***(score 1-5)***	**General Notes**

EXAMINER NAME DATE:_
